# Temperature gradients assist carbohydrate allocation within trees

**DOI:** 10.1038/s41598-017-03608-w

**Published:** 2017-06-12

**Authors:** Or Sperling, Lucas C. R. Silva, Aude Tixier, Guillaume Théroux-Rancourt, Maciej A. Zwieniecki

**Affiliations:** 1 Institute of Plant Sciences, Agricultural Research Organization, Gilat, 85280 Negev Israel; 2 Department of Plant Sciences, University of California Davis, Davis, CA 95616 USA; 3 Environmental Studies Program, Department of Geography, Institute of Ecology and Evolution, University of Oregon, Eugene, OR 97403 USA

## Abstract

Trees experience two distinct environments: thermally-variable air and thermally-buffered soil. This generates intra-tree temperature gradients, which can affect carbon metabolism and water transport. In this study, we investigated whether carbohydrate allocation within trees is assisted by temperature gradients. We studied pistachio (*Pistacia integerrima*) to determine: (1) temperature-induced variation in xylem sugar concentration in excised branches; (2) changes in carbon allocation in young trees under simulated spring and fall conditions; and (3) seasonal variability of starch levels in mature orchard trees under field conditions. We found that warm branches had less sugar in perfused sap than cold branches due to increasing parenchyma storage. Simulated spring conditions promoted allocation of carbohydrates from cold roots to warm canopy and explained why starch levels surged in canopies of orchard trees during early spring. This driving force of sugar transport is interrupted in fall when canopies are colder than roots and carbohydrate redistribution is compartmentalized. On the basis of these findings, we propose a new mechanistic model of temperature-assisted carbohydrate allocation that links environmental cues and tree phenology. This data-enabled model provides insights into thermal “fine-tuning” of carbohydrate metabolism and a warning that the physiological performance of trees might be impaired by climatic changes.

## Introduction

The effects of photoperiod on seasonal tree growth and phenology have been extensively studied and are known to be associated with changes in carbohydrate allocation in trees adapted to temperate climates^1, 2^. However, the role of temperature variability - daily or seasonal - on patterns of carbohydrate allocation remains unexplored^3^. Unlike day length, seasonal temperature regimes are strongly influenced by recent changes in global climate. In many regions, rising temperatures have inhibited tree growth, reduced yields of tree crops, and increased susceptibility of trees to biotic and abiotic stressors^4–6^. These negative effects could be attributed to a temperature-induced metabolic response that assists shifts in carbohydrate allocation within trees^7^. Specifically, in plants that experience high internal thermal variability, temperature affects cellular starch synthesis and degradation^8^, and respiration rates^9^. At the intercellular level, temperature fluctuations also affect membrane permeability and transporters’ activity, which in turn regulate the symplast to apoplast interactions^10^. Notably, trees do not experience one single temperature regime, but rather they dwell in two thermally different environments: highly variable air temperature that affects the canopy, and buffered (slow changing) soil temperature around the roots. With daily and seasonal climatic fluctuations this generates thermal gradients along the tree axial distribution paths (xylem and phloem), which drive the distribution of non-structural carbohydrates (NSCs).

A seasonal climate is characterized by prominent changes in temperatures around the year. Trees respond to these changes by relocating NSCs, which is externally manifested as phenological cycles^11^. Unlike annual plants that deplete their NSCs reserves within a single night and restore them the following day^12^, trees have large NSCs storage capacity, which under normal circumstances maintains a net positive annual carbon budget^7^. In mediterranean and temperate climates, a crucial function of this annual budget is that in spring, deciduous trees require abundant carbohydrates for vegetative and reproductive growth before their new leaves are productive. Afterwards, in summer, the canopy restores its carbohydrates storage in the form of starch to support essential metabolism during fall, winter’s dormancy, and the following spring. Trees also rely on starch reserves to cope with seasonal stresses and deficiencies, e.g. drought^13^, frost^14^, and pathogens^15^. Under severe stress, storage is especially important because assimilation of new carbon can be significantly limited^16^. Yet NSCs are not always stored where they are used. NSCs travel daily and seasonally between the tree’s compartments, but the role of intra-tree thermal gradients in governing this process has yet to be investigated.

At any given day, pools of NSCs undergo a dynamic cycle^17^ that ties biological cell processes to whole plant NSCs allocation^18^. Newly formed carbohydrates are exported from leaves to sites of growth or stored locally along the pathway. Generally, carbohydrates are distributed in the soluble phase (mainly as non-polar di-saccharides or tri-saccharides) and later used for respiration and growth, or they are stored as starch in wood and root reservoirs^7^. Phloem provides the long-distance pathway for sugars transport through the symplast. This is, however, a leaky system that allows continuous diffusion of sugars to the apoplast^19^ and can later feed the xylem parenchyma cells with NSCs for storage. Interestingly, the symplast ‘leakiness’ may form the basis for versatility in NSC redistribution within the dendritic system. It could enable trees to utilize carbohydrates according to their seasonal requirements in a two-direction transport system that slowly moves concentrated sugars in the phloem downwards while delivering diluted sap through the xylem to distally located branches. Research in this topic originated from farmers’ interest in increased productivity of tree crops and was recently evoked to explain tree mortality in forest ecosystems due to warming climates^20^. Yet the effect of the environment on NSCs redistribution is not well understood. Even the most elaborated models of carbon ‘sinks’ and ‘sources’ within trees lack explanations on what would initiate the transport. Local carbohydrates deficiencies (due to emerging buds, developing fruits, or extending branches) are, by virtue of their size and low photosynthetic capacity, unlikely to initiate carbohydrates accumulation near the sites of future growth observed in branches^11^. For instance, in spring, even before bud-break, branches are reported to accumulate carbohydrates^21^, while phloem is presumed to be non-functional^22, 23^.

Based on recent theoretical developments^3^, we hypothesize that NSC storage, release, and allocation are assisted by temperature gradients along transport pathways of temperate trees. This hypothesis is an extension of our former conceptual model which proposes that internal temperature gradients in trees assist diurnal and seasonal carbon allocation and take part in regulating phenological cycles. If proven correct, this mechanism could explain coordinated carbohydrate redistribution in trees and upscale our discussion from days to seasons. Our proposal is consistent with observations of enzymatic thermal kinetics, i.e. that cold environments promote release of sugars and warming leads to starch accumulation. We specifically hypothesize that trees would release soluble carbohydrates from cold parts and accumulate starch in warm parts. To test this hypothesis experimentally we set out to determine the effect temperature on uptake and release of carbohydrates from stem parenchyma cells in excised branches. Next, using stable isotope (^13^CO_2_) labeling experiments, we quantified the fate of new photosynthates in saplings under controlled root-to-canopy temperature regimes. Finally, we determined internal temperature gradients in mature trees under field conditions and empirically tested relationships between carbohydrate allocation and tree phenology to develop a new mechanistic model of temperature-assisted carbon allocation within trees.

## Results

### Experiment #1 – The effect of temperature on xylem sap sugar concentration

**Figure 1:**
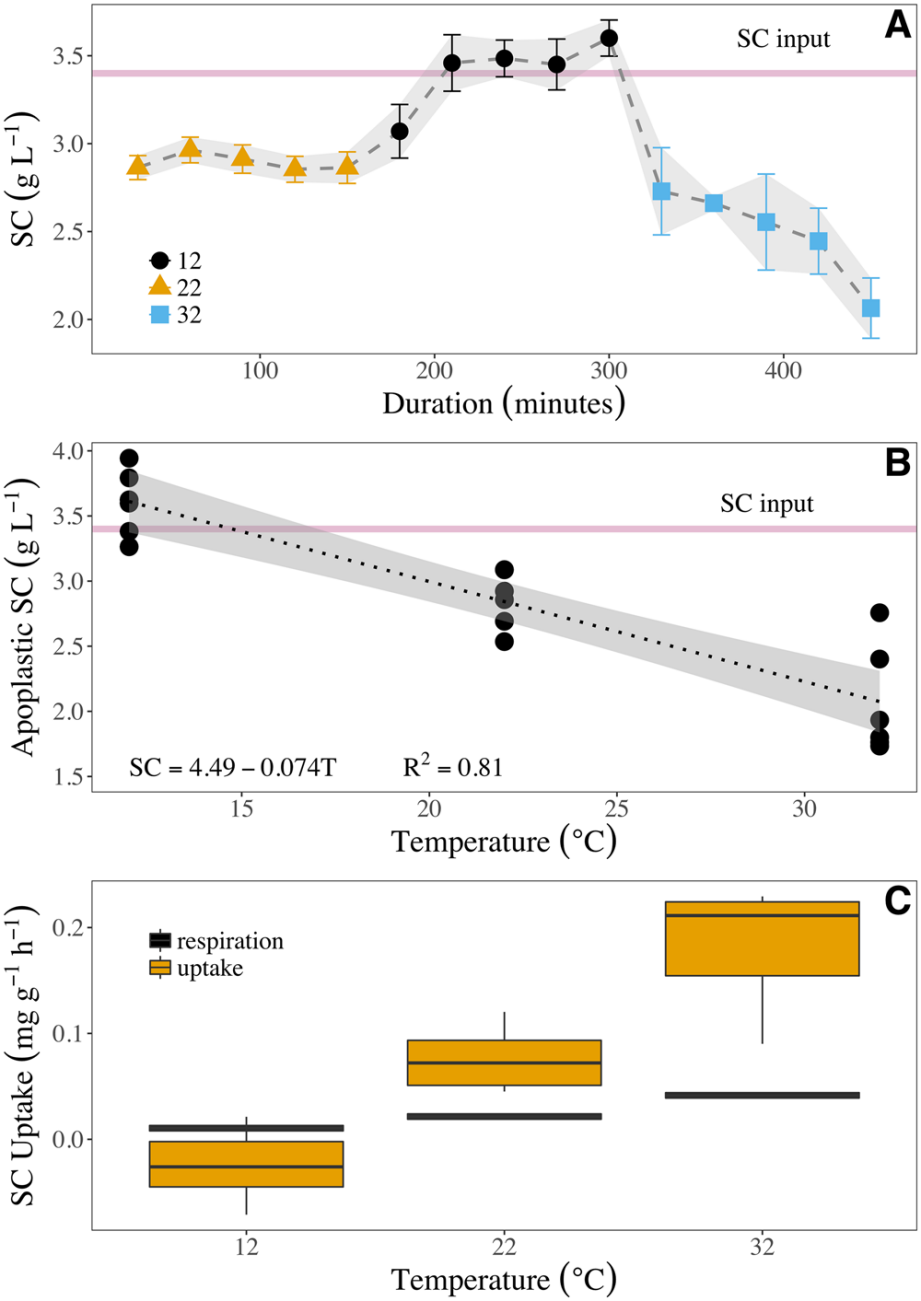
Apoplastic SC concentrations in perfused branch segments at variable temperatures. 15 cm long and 10 mm in diameter branches were submitted to 12 °C (blue circles), 22 °C (green triangles), and 32 °C (red squares) and perfused with sucrose solution. The solution’s input concentration was 3.42 g L^−1^ sucrose (red horizontal line) and flow rate was 0.68 g h^−1^. (**A**) SC concentration of perfused solution in branches changing temperature every 150 minutes (from 22 °C to 12 °C and then to 32 °C, 450 minutes total). (**B**) The final SC concentration of perfused solution (after 150 minutes at a given temperature) decreased with temperature in a linear manner (dotted line, R^2^ = 0.81, df = 16, p = 1.433e^−7^, grey area denotes 95% confidence intervals). The intercept value of initial SC concentration (i.e. the temperature at zero net uptake of SC) was 14.5 °C and is referred to as the compensation point. (**C**) The rate of SC uptake from the perfused solution (orange boxes) vs. the rate of SC lost to stem respiration (black panels) during the 150 minutes at each temperature.

The concentration of soluble carbohydrates (SC) in the solution perfused through *P*. *integerrima* excised branch segments was significantly affected by temperature (Fig. 1A). The sugar concentration of the sap perfused through stems at 22 °C was relatively constant during 2.5 hours, ca. 2.9 g L^−1^ which is 0.5 g L^−1^ less than the input concentration (P < 0.05). When the same branch segments were cooled to 12 °C the sap sugar concentration levels increased above the input, reaching 3.55 g L^−1^ on average, or 0.13 g L^−1^ more than the input concentration (P < 0.05). Conversely, when the same branch segments were heated to 32 °C they became a strong net sink for sugar; i.e., significantly (P < 0.05) reducing the sugar concentration in the perfused solution to 2.1 g L^−1^, or 1.32 g L^−1^ less than the input concentration. A least square regression between stem temperature and final SC concentration in perfused solution (Fig. 1B; $SC=4.49-0.074\times T$, R^2^ = 0.81) shows that at 14.5 °C (±2.2 °C) net SC uptake by cells would be zero, i.e. the rate of carbohydrate use and storage equals the rate of mobilization of stem reserves (from starch digestion or cellular unloading of SC). Thus, 14.5 °C is referred to as the compensation point for cellular sugar uptake. The increasing rate of SC uptake from the solution with increasing stem temperatures was reflected in the amount of carbon lost through respiration (82, 149, and 272 µmol CO_2_ m^−3^ s^−1^ on average at 12 °C, 22 °C, and 32 °C, respectively). However, the thermally-induced SC uptake was much larger than the amount of respired carbon. Respiration accounted for carbon losses of 0.013, 0. 024, and 0.044 mg g^−1^ h^−1^ at 12 °C, 22 °C, and 32 °C, respectively, whereas the rates of carbon uptake were −0.02, 0.08, and 0.18 mg g^−1^ h^−1^ at the same temperatures (Fig. 1C). In other words, the dilution of the apoplastic solution in branch segments at 22 °C and 32 °C was about 4 times the respiratory demands and, thus, SC uptake is interpreted as a direct measurement of increased SC storage in the stem.

### Experiment #2 - The effect of temperature gradients on carbohydrate allocation

**Figure 2:**
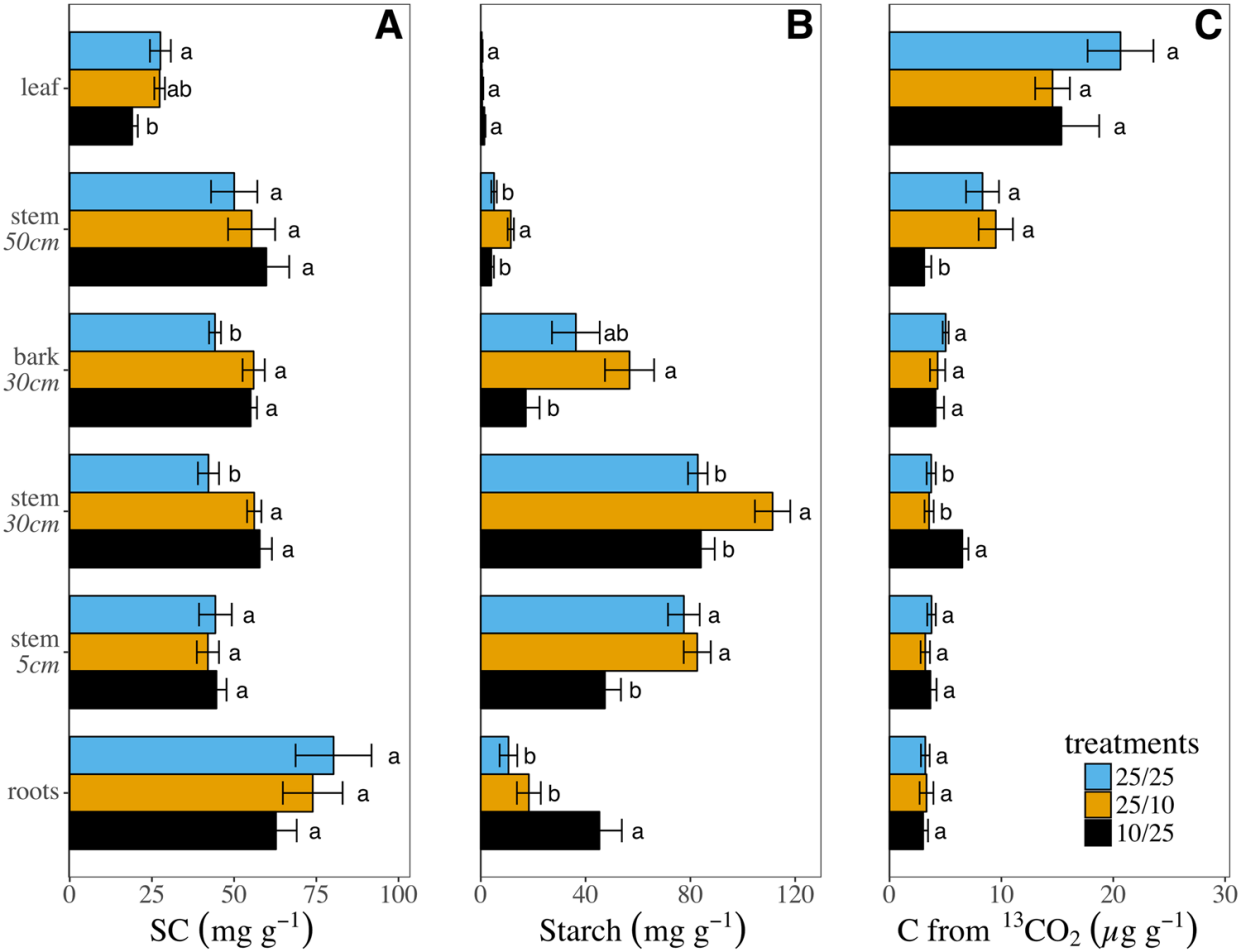
Impact of temperature gradients on nonstructural carbohydrates redistribution in young trees. (**A**) Soluble carbohydrates (SC), (**B**) starch, and (**C**) C derived from ^13^CO_2_ levels in the leaves, bark at 30 cm from soil, stem at 50, 30, and 5 cm above soil, and roots control trees (25 °C_shoot_/25 °C_root_, light gray columns), simulated spring (25/10, gray columns), or simulated fall (10/25, dark gray columns). Error bars denote standard errors and lowercase letters denote statistical differences (two-ways Anova and Tukey-HSD, P < 0.005, df = 16).

Analyses of young trees in a greenhouse setting showed baseline SC average concentrations of about 27 mg g^−1^ in leaves, 50 mg g^−1^ in the stem, and 72 mg g^−1^ in roots (Fig. 2A). This baseline distribution shifted significantly in response to temperature treatments. In leaves, SC decreased (19 mg g^−1^; p < 0.05) if the canopy was colder than the soil (i.e., 10 °C/25 °C treatment – simulated fall) relative to 25 °C/25 °C controls. SC in the bark and the stem at 30 cm increased in both simulated spring (25 °C/10 °C) and fall treatments by 14 and 16 mg g^−1^, respectively. Starch was nearly negligible in leaves, but increased towards the stem (reaching 93 mg g^−1^) and decreased to 25 mg g^−1^ in roots (Fig. 2B). If the canopy was warm relative to the soil (i.e. 25 °C/10 °C) starch accumulated in the upper stem (50 cm–11 mg g^−1^), bark (57 mg g^−1^), and lower stem (111 mg g^−1^). In a cold canopy on the other hand (i.e. the 10 °C/25 °C treatment) starch decreased in the bark (17 mg g^−1^) and in the lower stem (47 mg g^−1^ at 5 cm from the soil), while at the same time accumulating in the roots (45 mg g^−1^). Following a pulse of ^13^CO_2_, the ^13^C recovered in each compartment, which denote the presence of new photosynthates, reached 17 µg in leaves. The amount of ^13^C decreased in the upper stem to 7 µg and leveled at 4 µg in all the other tree compartments (Fig. 2C). Yet, the amount of ^13^C decreased to from 8 to 3 µg in the upper stem and accumulated from 3.5 to 7 µg in the upper stem (at 30 cm) if the canopy was cold (10 °C/25 °C treatment) compared to control. This implies that the carbohydrates redistribution in the 10 °C/25 °C treatment was compartmentalized, i.e. NSCs did not travel between the cold crown and the warm roots. Thus, SC in the canopy was enriched with ^13^C while the starch that accumulated in the roots was not.

### Field observations

**Figure 3:**
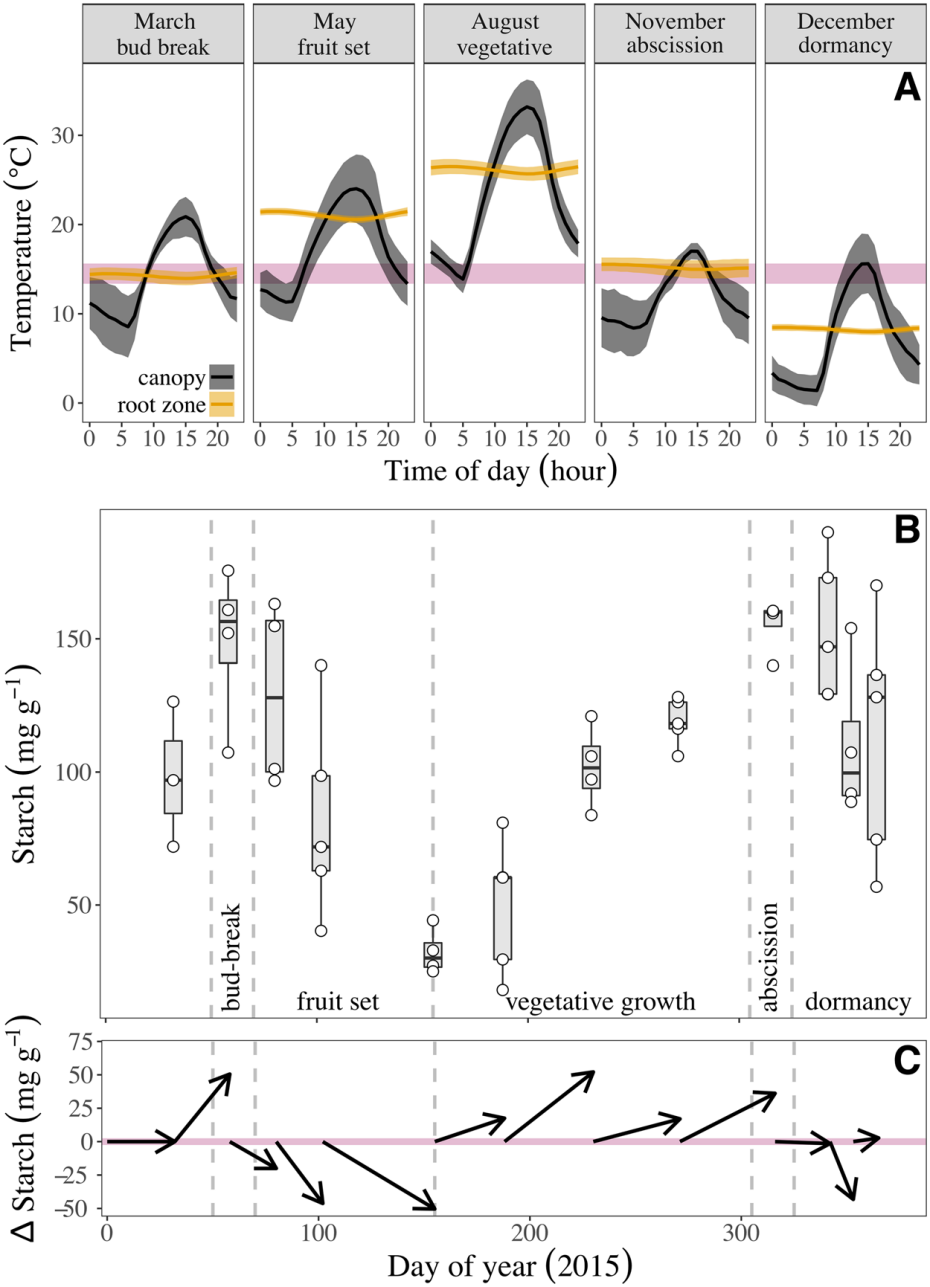
*In*-*situ* representation of seasonal changes in root-to-shoot temperature gradients and starch levels in the canopy of mature orchard trees. (**A**) Temperature variation and gradients during bud-break, fruit set, vegetative growth, abscission, and dormancy at the canopy (blue line, shaded area denotes SE values of 7 days) and in the roots zone (30 cm deep, red line and shade). Pink line represents the 14.5 °C compensation point (established in experiment #1 with the appropriate 95% confidence intervals). (**B**) Starch levels in one-year-old branches collected at 11:00 from 5 mature trees. Gray boxes denote the 95% confidence intervals, thick line in the box exhibits the average, and circles show the starch levels at each tree. Dashed vertical lines separate between the phenological stages. (**C**) A vector illustration to emphasize the magnitude and direction of starch dynamics in the branches at the different phenological stages.

Supporting our experimental data, dynamic gradients between air and soil temperatures in mature trees under field conditions caused measurable shifts in the direction and magnitude of the intra-tree thermal regimes during the course of days and seasons (Fig. 3A). In spring, when the buds break, the root zone is still cold (ca. 13 °C at 30 cm below ground) while the canopy gradually warms to 22 °C during midday. During most of the day (10:00 to 16:00) we measured air temperatures well above the stem compensation point reported in experiment #1 (14.5 °C, Fig. 1B). Under these conditions sugars should move towards the canopy (according to experiment #2). Indeed, we observed significant accumulation of starch in branches (see Fig. 3B for raw values and Fig. 3C for net direction of starch content in relation to the previous sampling). During a short time preceding bud-break starch levels increased from ca. 96 mg g^−1^ DW (constant level during dormancy) to 135 mg g^−1^ DW while reserves were the only possible source of NSCs (i.e. leaves were still absent). Later in the growing season both soil and air warmed above the equilibrium temperature. At this time the governing processes were most likely related to fruit growth, which depleted the starch storage to 30 mg g^−1^ DW (DOY 150), followed by high photosynthetic productivity during the summer, which restored the starch pools back to 110 mg g^−1^ DW (DOY 285). In the fall (October), soil temperature remained above the 14.5 °C compensation point (see experiment #1) but the canopy was cold (10 °C at night), which should block SC transport downwards (according to experiment #2). Indeed, starch levels stabilized at the time of senescence as canopy ceased to deliver NSCs to roots (Fig. 3B and C). The gradual decrease from ca. 110 mg g^−1^ (end of summer) to 90 mg g^−1^ (early spring) could therefore be attributed either to translocation of NSCs from branches to main stem (as suggested by experiment #2) or to canopy respiration in winter. Finally, during winter (dormancy) both soil and air were too cold for NSCs translocation, which led to slow and localized starch exploitation in the canopy.

## Discussion

Our results support the hypothesis that internal temperature gradients affect the distribution of carbohydrates in trees and influence phenological patterns. The goal of this study was to determine if temperature influences the parenchyma cells potential to store (starch synthesis) or release SC (starch degradation) (experiment #1) and to evaluate how root-to-canopy temperature gradients affect the fate of new photosynthates within trees (experiment #2). In temperate climate zones, temperature varies daily and seasonally, with soil and air exhibiting measurable thermal differences that rarely converge in either timing or magnitude. By virtue of being poikilothermic (i.e., having variable internal temperatures) and spanning both soil and air environments, trees growing in temperate regions frequently experience internal thermal gradients. These gradients vary seasonally and affect NSCs metabolism by altering the demand for carbohydrates, their transport, and their synthase rates^3^. Our work confirms the hypothesis that NSCs storage in a model deciduous tree species is altered by the internal thermal variability, along the carbon transport pathway, assists axial NSCs allocation in a predictable way.

Based on the results from experiment #1, we concluded that the compensation point for carbohydrate exchange between xylem parenchyma cells and apoplast is at 14.5 °C. Above this temperature parenchyma cells accumulate sugars, storing the surplus as starch. Below 14.5 °C cells release SC, degrading starch and effectively unloading SC to the xylem. It is important to note that plants don’t need to ‘sense’ temperature for this shift in activity. It most likely results from the differences in thermal coefficients between the starch synthesis and the starch degradation pathways^8^. The phloem transport is ‘leaky’ and interacts with adjacent cells and conduits^19^, enabling further diffusion of NSCs to the apoplast and possibly carbohydrate transport through the xylem. This behavior is expected from tree organs that support flows by integrating conduits and living parenchyma cells, e.g. roots, stem, and branches, and show corresponding tendency to accumulate SC under cold conditions^24, 25^. However, only small stem segments fit this type of inquiry and to broaden our insights we needed to study these mechanisms at the whole tree level.

**Figure 4:**
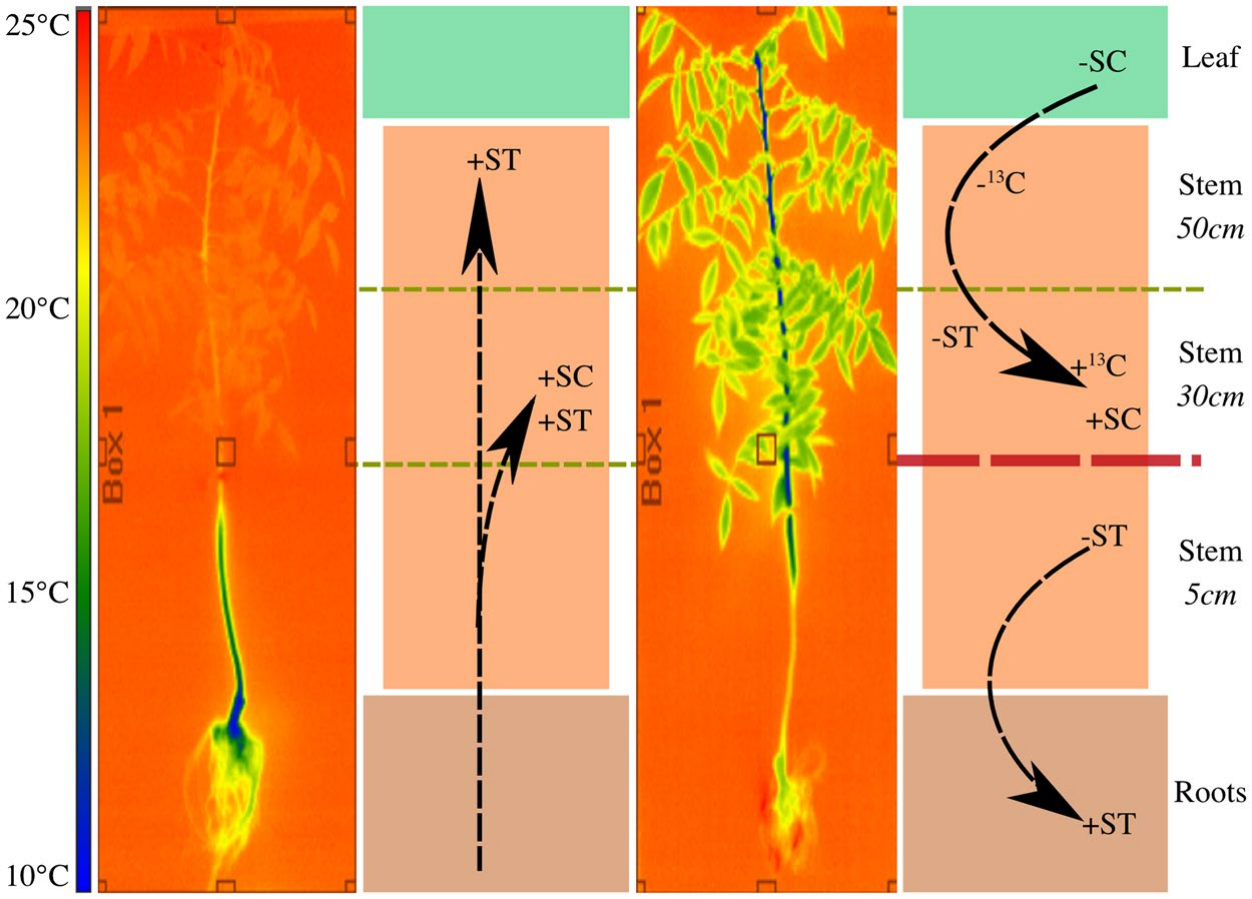
A conceptual model of carbohydrate redistribution due to root-to-canopy temperature gradients. The model summarizes differences observed in experiment #2 between the 25 °C_shoot_/10 °C_root_ (spring) and 10 °C_shoot_/25 °C_root_ (fall) treatments and the control group (25 °C_shoot_/25 °C_root_). Significant changes (relative to control) in soluble carbohydrates (SC), starch (ST), or ^13^C derived from new photosynthates following a pulse of ^13^CO_2_ are denoted by ± signs. Arrows represent the proposed path for sugar redistribution and the horizontal red line shows an impaired pathway and compartmentalization of NSC transport. Thermal images of the tree demonstrate the temperature gradients from root to canopy.

In experiment #2 we scaled-up our investigation to show that intra-stem temperature gradients assist and may even temporarily control the direction of NSCs transport in trees. Trees with roots colder than 14.5 °C and a warm canopy (i.e. a thermal regime that roughly represent a spring day) accumulated NSCs in the warm part of the stem (summarized in Fig. 4 and consistent with experiment #1). However, there was no change in carbon content derived from the ^13^CO_2_ pulse, which means that these NSCs came from storage in the lower parts of the tree and not from new photosynthates^26^. NSCs traveling upwards require either reversal of phloem flow – an unlikely scenario – or sugar transport through the xylem. Our results support the notion that as temperature gradients change from winter to spring, xylem redistributes stored NSCs. This NSCs supply upwards is crucial to support the canopy during bud-break, flowering, and vegetative growth before the leaves are productive. We observed this accumulation of NSCs in branches of matured *P*. *integerrima* prior to bud-break (Fig. 3C), which is consistent with previous studies^11, 22^, showing that intra-tree temperature gradients facilitate the distal translocation of sugars through the xylem.

The alternative temperature scenario we investigated, i.e. trees with canopy colder than 14.5 °C and warmer roots, simulated the conditions they often experience in late fall. In these conditions, we observed a compartmentalization of carbohydrate redistribution between canopy and roots. Our isotopic measurements showed that cold canopies withheld the export of new photosynthates to the warm roots and, as a result, accumulated high levels of NSCs in the upper stem (Figs 2 and 4). The warm stem (lower part) and roots maintained downward oriented transport and drained NSCs towards the deeper roots. The lack of upward xylem transport of NSCs is most likely attributed to the low transpiration of the cold canopy, which matches trees phenology in late fall. These findings suggest that trees stop NSCs redistribution between their canopy and roots prior to entering dormancy, a process that might trigger accumulation of starch in the stem near the canopy for winter protection mechanisms and supply reserves for spring bud-break^27^. The slow release of SC from the lower stem and large roots reserves toward the deeper roots most likely supports the common winter roots-growth of trees in seasonal climate^22^.

Our experimental results and the conceptual model of carbohydrates redistribution in early spring and late fall (Fig. 4) were confirmed by field observations of carbohydrate content in branches of *P*. *integerrima*. At that time the trees were not photosynthetically active and storage played a crucial rule in their performances^28^. We observed corresponding starch accumulation in the canopy towards spring and a gradual depletion through winter, as demonstrated in experiment #2. Additional starch could have been synthesized from local SC pools, but an extensive review of the literature in this field suggests that sugar levels don’t change to that extent^29^. Furthermore, in our isotope labeling experiment under ‘simulated spring’ (25 °C/10 °C), we found accumulation of NSCs that did not originate in the canopy. This can be concluded with great confidence because NSCs originating from the canopy were consistently enriched in ^13^C (>3-fold higher than root or low stem reservoirs, Fig. 3C). Therefore, the whole tree analysis indicates that upward movement of NSCs was induced solely by the thermal gradient. Further support for this conclusion stems from the fact that in the ‘simulated fall’ treatment the reverse response occurred, i.e. NSCs originating from the canopy (and thus enriched in ^13^C) accumulated in the upper stem as a result of the inverted temperature gradient. Thus, in the case of the field experiment, we can also conclude that NSCs likely traveled in spring from distal storage locations where temperatures are significantly cooler, i.e. the roots. This regulation of NSCs allocation in trees during bud-break and leaf abscission by internal spatial temperature variability implies that climate change will negatively impact tree growth, phenology, and reproduction. Globally, the forecasted trend of climate change is towards higher temperatures at nights and throughout winters^30, 31^ while spring timing is expected to become more variable^32^. Based on our experimental results, widespread changes in carbohydrate allocation should be expected in trees adapted to temperate climates. These changes would include disrupted reproduction cycles in spring and warming-induced increases in mortality during harsh summers and winters. Evidence of this have already been documented in forest ecosystems^33–35^ and tree crops^36^, but these studies were not able to identify the underlying physiological mechanisms of tree growth decline.

## Conclusion

The present study identifies the role of thermal gradients in carbon balances of trees and provides a simple explanation for recent reports of shifts in plant physiology due to climate change. For example, our results could explain asynchronous flowering in recent springs by impaired access to roots’ NSC pools in warmer soils^37^. Furthermore, this study could be extended to include the effect of winter precipitation patterns on root temperature, as moisture affects thermal conductivity in soils^38^. In controlled environments, intra-plant temperature gradients should also be taken into account as they could explain well-described differences in growth between greenhouse and field plants^39^. Finally, our findings could explain how shifts in temperature increase trees’ vulnerability to pathogens^40^, drought, or heat waves^13^, as a result of increasing reliance on carbohydrates storage^41^. It is possible that as trees’ internal temperature gradients, altered due to a changing climate, can result in asynchronous phenological cycles and increased susceptibility to other abiotic and biotic stressors.

It is important to note however that intra-tree temperature gradients are not the main driver of NSCs transport and our conclusions should not be extrapolated to all seasons. We demonstrate that in our research site (field observation) both roots and canopy were warmer than the compensation point (14.5 °C) during the summer and temperature gradients were not an effective driver for NSCs transport in such conditions. In this case, the release of NSCs to the xylem is probably limited and the stem becomes a net carbohydrate sink, as described in previous studies^11, 21^. Moreover, the starch levels in branches we measured during summer are influenced by the phenological stage at time of sample collection, e.g. fruit development, vegetative growth, or storage restoration. Moreover, the compensation point for NSCs cellular uptake may change seasonally as enzymatic pathways undergo biological changes^3^ with changes of enzymes’ isoforms that change their thermal coefficients^8^. It is also likely that this compensation point is species-specific and represents the legacy of adaptation to past environments. However, our findings are supported by previous research on other tree species which have described xylem loading with SC in spring [e.g. sugar maple^24^, walnut^42^, and birch^43^]. In light of these findings and our experimental results, we argue that as trees cool below the compensation point during emergence from dormancy, cells degrade starch and release SC to the apoplast. At night there is relatively lower transpiration, especially in winter, and SC accumulates in the xylem. As the days in late winter are already warmer the temperature gradients between roots and canopy facilitate xylem transport of these soluble NSC upwards. This transport is probably further supported by osmotic gradients formed due to spatial differences in SC concentrations. Once NSCs reach the warm canopy they are synthesized to starch and support bud break and the emerging leaves through spring.

In conclusion, trees respond to root-to-canopy temperature gradients by changing their local NSCs management and tree level redistribution. This predictable response depends on symplast-apoplast SC equilibrium, effectively reflecting the role of parenchyma cells in the NSCs balance and the potential for xylem to transport SC. Xylem redistribution of NSCs is important during periods of low transpiration and intensive biological activity that require redistribution of carbohydrates, e.g. bud-break in spring or leaf senescence in fall. At these times, the intra-tree temperature gradients are especially prominent yet they point to opposite directions – in spring the roots are cold and the canopy is warmer (implying allocation of NSCs from the roots to the warmer canopy) while in fall the roots remain warm and canopy temperatures decline (suggesting slow shift of NSCs from canopy to roots).

## Methods

### Tree selection

We conducted the experiments using a well-studied and economically relevant tree species in California’s agriculture, the pistachio’s rootstock, *Pistacia integerrima* (J. L. Stewart ex Brandis). This was an ideal model for our experiments because species of the genus *Pistacia* are adapted to mediterranean climates, native to many temperate seasonal habitats, and thus thrive in environments with large soil-to-air temperature gradients. This species is also abundant in natural forests and is an important source of rootstocks for agriculture^44^. Given its typical deciduous phenology and economic importance in farming -1.6 billion dollars industry in the US alone in 2014^45^ - the results of this research are relevant to both the scientific and agricultural communities.

### Experiment #1 – Testing the effect of temperature on xylem sap sugar concentration

We studied the effect of temperature on xylem sap sugar concentration in excised stem segments. In our setup only one parameter was changed – temperature. Xylem sap flow rate was constant and in range of typical transpirational flow rates for the given branch size. Branches from 6 trees was collected (6 branches total, UC Davis research farm: 38.5°N, −121.79°W) in September 2015 and directly transported to the laboratory. 15 cm long stem segments (ca. 10 mm diameter) were excised from the branches underwater and connected to nylon tubing filled with a 3.42 g L^−1^ sucrose solution (10 mM). We flushed manually (with a syringe) the stems with 15 mL of the sucrose solution and then fixed one end to a peristaltic pump that continued to perfuse the stem with the sucrose solution at 0.68 g h^−1^ (the approximated transpiration rate for branches of that size). During perfusion, all branches were kept for 150 minutes at each of the following temperature (consecutively): 22 °C, 10 °C, and 32 °C. We collected sap from the open end of the branches every 30 minutes and kept the sap in 4 °C until analysis of sugar content (method described below). Adjacent branches from the same trees were collected for respiration measurements in a sealed chamber and a gas analyzer (Li-6400XT, LiCor Biosciences, Lincoln, NB) in temperatures between 0 °C and 30 °C to derive its kinetics. Then we calculated the NSC requirements of the branches during our experiment as: $NSC={R}_{(T)}\times t\times V$, where *R*_(*T*)_ is the respiration as a factor of temperature, *t* is the duration of our experiment (150 minutes; each temperature is treated independently), and *V* is the branches’ volume.

### Experiment #2 – Testing the effect of temperature gradients on carbohydrate allocation

We used 18 two-year-old *P*. *integerrima* clonal saplings cultivated under field conditions at the horticulture research facility of UC Davis. On September 10^th^ (2015), at 16:00, we exposed the trees to air with enhanced levels of ^13^CO_2_ in ambient light for one hour in a closed chamber. At 17:00 we moved the trees to dark rooms for 15 hours, where the temperature treatments were applied (see following paragraph for details on the enrichment procedure). Darkness of 15 hours reflects the effective time of non-photosynthetic activity in *P*. *integerrima* in fall. *P*. *integerrima* is a perennial plant with large starch reserves and there was no risk of temporal starvation. The longer darkness also promoted homogeneity of ^13^C incorporation to starch granules. We placed the pots in an insulated and temperature-controlled box that also covered 15 cm of the stem. In this way, temperatures in the roots (soil) were independent from the canopy (air). We applied 3 temperature gradients to 6 trees per treatment (T_air_/T_soil_: 25 °C/25 °C, 25 °C/10 °C, and 10 °C/25 °C). These temperature gradients were selected to represent summer, spring, and fall conditions, respectively. We continuously monitored the temperatures around the canopy and roots with *k*-type thermocouples to verify the presence of axial thermal gradients. The next day at 08:00 we harvested the trees and collected leaves, bark from 30 cm above soil, stem sections (50, 30, and 5 cm above the soil), and roots samples for ^13^C and carbohydrate analysis. The bark was removed from stems for xylem tissue analysis. We dried the tissues at 75 °C to constant mass, ground the samples, and analyzed the dry powder for soluble carbohydrates (SC), starch, and ^13^C isotopic enrichment. We refer to the 25 °C/25 °C treatment as *reference* and present the differences in carbon levels caused by the 25 °C/10 °C and the 10 °C/25 °C treatments.

### Isotopic labeling

The ^13^C enrichment experiment followed methods developed by Silva *et al*.^46^. Briefly, we used a sealed clear chamber with a fan inside to circulate air under ambient sun light. Trees from all treatments (6 per treatment – 18 total) were simultaneously put into the chamber before a pulse of CO_2_ at 98% ^13^C enrichment was injected. The labeled CO_2_ was generated using barium carbonate (Ba^13^CO_3_; Sigma-Aldrich) gently mixed in sulfuric acid solution, measured to generate an initial CO_2_ concentration in the chamber of ca. 600 ppm. Gas aliquots were taken regularly for the duration of the labeling event (one hour) from a small port in order to monitor the absorption of gases by plants. The temperature during the labeling event was ca. 25 °C and previous tests showed that the CO_2_ leakage of the empty chamber during two hours of observation was negligible (<20 ppm). During the experiment, when plants were in the chamber, the concentration of CO_2_ decreased by approximately 150 ppm over the course of the hour, but remained above ambient concentrations and thus did not become limiting. After one hour in the labeling chamber, we moved all plants to a controlled environment facility and imposed the temperature gradient treatments as described above. Isotopic analysis of leaves, stem segments, and roots before and after labeling were used to calculate the amount and allocation of newly photosynthesized compounds derived from the ^13^CO_2_ pulse following standard protocol^47^. Measurements of ^13^C were conducted using an Elementar Vario EL Cube or Micro Cube elemental analyzer (Elementar Analysensysteme GmbH, Hanau, Germany) interfaced to a PDZ Europa 20–20 isotope ratio mass spectrometer (Sercon Ltd., Cheshire, UK) at the Stable Isotope Facility of the University of California, Davis. Uptake and allocation of labeled carbon was expressed on the basis of mass, as excess ^13^C (μg g^−1^) relative to baseline control values determined for the same plants before the labeling event. We evaluated the initial homogeneity of ^13^C enrichment in stem’s segments at 5 and 30 cm above the soil after 1 hour of ^13^CO_2_ application. We cut 6 trees right after the labeling, found that initial enrichment was similar both heights and repeated measurements at those heights in other trees for redistribution analysis.

### Analysis of starch and soluble carbohydrates

We modified a starch hydrolysis assay^48^ to quantify starch in our plant samples. We ground dry tissue in a ball grinder (MiniBeadbeater-96, Glen Mills Inc., NJ). Tissue samples of 25 mg were mixed with 1 ml of DI water and incubated for 15 minutes in a water bath at 72 °C. After a centrifugation of 10 minutes at 21000 g, we collected 50 µL of the supernatant for SC analysis (see below). We then washed the pellet twice more (in 1 ml DI water) and added to it 500 µl of Na acetate buffer (0.2 M, pH 5.5), 100 µl amyloglucosidase (70 units ml^−1^, Sigma-Aldrich), and 100 µl amylase (7 units ml^−1^, Sigma-Aldrich). After mixing, samples were incubated 4 hours at 37 °C for full enzymatic starch digestion, and then centrifuged at 21000 g for 10 minutes. Finally, 50 µL of supernatant were collected for analysis of the digested soluble sugars in liquid samples. Soluble carbohydrates in the liquid samples (perfused sap collected in experiment #1 and prepared samples from experiment #2) were diluted 21 times and analyzed according to Leyva *et al*.^49^. In short, 50 µL samples were mixed with 150 µL of anthrone in sulfuric acid (0.1%, w/v) in a 96-well micro-plate. The plate was heated at 100 °C for 10 minutes, and then equilibrated to room temperature for 10 minutes. We determined the carbohydrates levels as glucose equivalents from the colorimetric reading (Thermo Scientific Multiskan) of absorbance at 620 nm (A_620_).

### Field observations

During 2015 we periodically (ca. once a month) collected branches from 5 *P*. *integerrima* trees (Wolfskill Experimental Orchards: 38.5°N, −121.97°W) at 11:00 and immediately placed them on dry ice in insulated containers and returned to the laboratory. This is a nondestructive application to quantify the immobile carbohydrates reserves (i.e. starch) in an essential part of the tree – the canopy, where buds will develop to fruits at the end of winter. Bark and wood were separated and dried for 48 hours in 75 °C and were analyzed for soluble sugars and starch contents as described above. Soil temperature in our field was measured at depth of 30 cm every minute during 2014 using a CR1000 datalogger (Campbell Scientific, Logan, UT, USA). Air temperature data were collected from the CIMIS data system (California Irrigation Management Information System). To create characteristic diurnal trends throughout the year at each phenological stage (i.e. bud break, fruit set, vegetative growth, abscission, and dormancy) we averaged hourly measurements for one representative week of each phenological stage, i.e. the 1^st^, 10^th^, 19^th^, 33^rd^, and 46^th^ week of the year.

### Statistical Analysis

We used R (R version 3.2.1, 2015, R Core Team) for all statistical analysis. For experiment #1 we analyzed the data using linear regressions to test the relation between apoplastic SC and temperature with 95% confidence intervals. For experiment #2 we used a two-way ANOVA and compared the results with the Tukey-HSD test.
